# Bilateral Ureteral Obstruction Caused by Stone Formation After Laparoscopic Appendectomy in Children

**DOI:** 10.1089/cren.2018.0052

**Published:** 2018-09-01

**Authors:** Ya-Fu Cheng, Yao-Jen Hsu, Tai-Wai Chi, Yu-Wei Fu

**Affiliations:** Division of Pediatric Surgery, Department of Surgery, Changhua Christian Hospital, Changhua City, Taiwan.

**Keywords:** ureteral obstruction, ureteral stone, pediatrics, appendectomy

## Abstract

In children, urologic complications are rare after laparoscopic appendectomy (LA). In this article, we report the case of a 9-year-old boy with bilateral hydronephrosis caused by ureteral calculi 10 days after he had undergone LA. The patient's urinary output normalized and renal function recovered after stone extraction and bilateral ureteral stent insertion.

## Introduction

Appendicitis is the most common surgical indication for acute abdomen in children; it occurs in 5.1% of all nonscheduled hospital visits. It most frequently presents in patients aged 10–19 years. Laparoscopic appendectomy (LA) is the standard treatment procedure for appendicitis. Common complications after LA for unperforated and perforated appendicitis, respectively, were ileus (2.18% and 14.08%), urinary tract infection (1.25% and 1.39%), pneumonia (0.44% and 1.44%), acute respiratory failure (0.44% and 1.54%), postoperative bleeding (0.32% and 0.37%), and acute kidney injury (0.41% and 1.78%). Urologic complications are uncommon, and only few studies have reported hydronephrosis and ureteral obstruction. In this article, we report the case of a 9-year-old boy who was diagnosed with acute appendicitis and underwent LA. Bilateral ureteral stones with hydronephrosis were observed 10 days postoperatively.

## Case Presentation

**FIG. 1.**
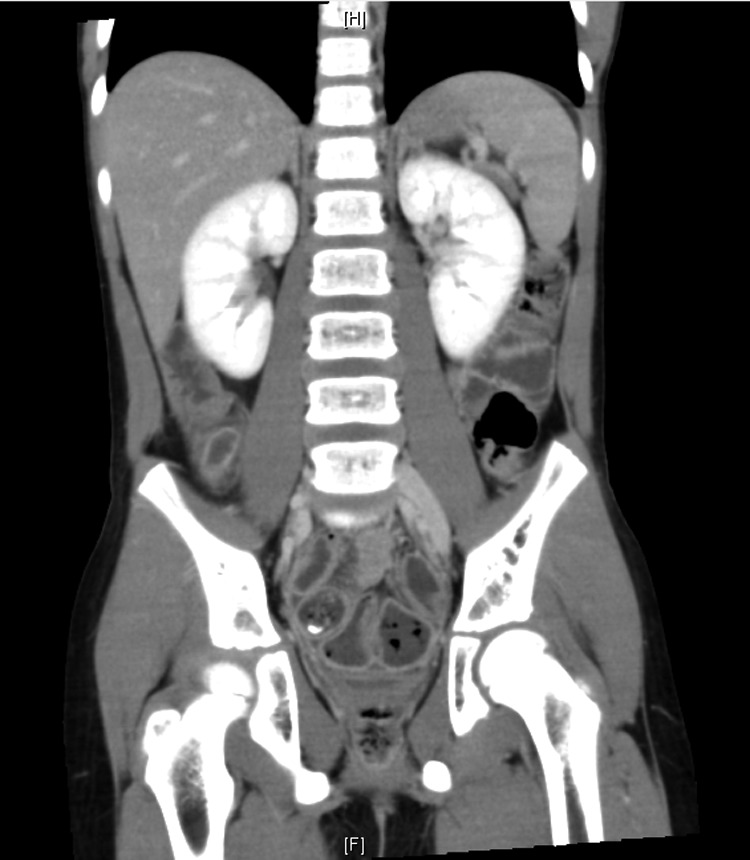
Abdominal CT revealed an engorged appendix with no hydronephrosis over the bilateral kidney.

A 9-year-old boy presented to our pediatric emergency department with abdominal pain. The pain was initially located at the periumbilical area and then migrated to the right lower abdomen. Fever for 2 days was reported. The patient had not experienced nausea or vomiting. His physical examination revealed abdominal tenderness over the right lower abdomen, without peritonitis. Laboratory analysis revealed a white blood cell count of 13,100 μL (range: 3500–9100 μL; neutrophilia, 84.9%) and creatinine level of 0.71 mg/dL (range: 0.70–1.30 mg/dL). Abdominal CT revealed a fecalith at the tip of the appendix and absence of hydronephrosis in the bilateral kidney (Fig. 1). Because acute appendicitis was suspected, the patient underwent LA. Acute perforated appendicitis with turbid diffused ascites was noted.

The patient received triple antibiotic therapy postoperatively (ampicillin, 1000 mg four times daily; metronidazole, 260 mg three times daily; and gentamicin, 50 mg two times daily). The early postoperative course was uneventful, with adequate urinary output of 2800 mL/day and white blood cell count of 9700 μL (neutrophilia, 67.5%). During the operation, ascites culture grew *Escherichia coli*, *Streptococcus constellatus*, and *Pseudomonas aeruginosa*. After observing good bowel function, well-tolerated diet, and reduced pain, outpatient follow-up was scheduled, and the patient was discharged.

**FIG. 2.**
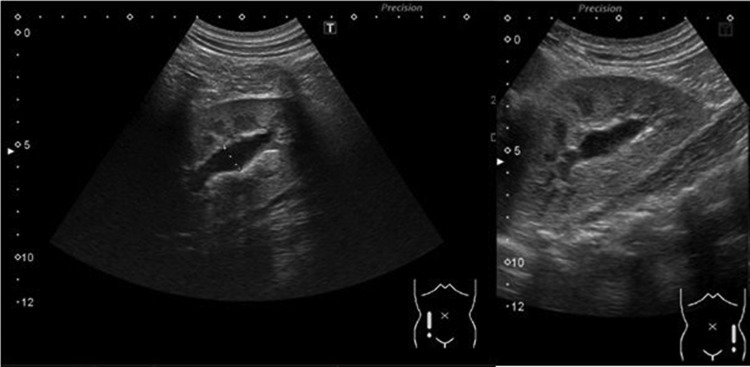
Renal ultrasonography revealed bilateral mild-to-moderate hydronephrosis ∼11 mm after laparoscopic appendectomy on POD10.

**FIG. 3.**
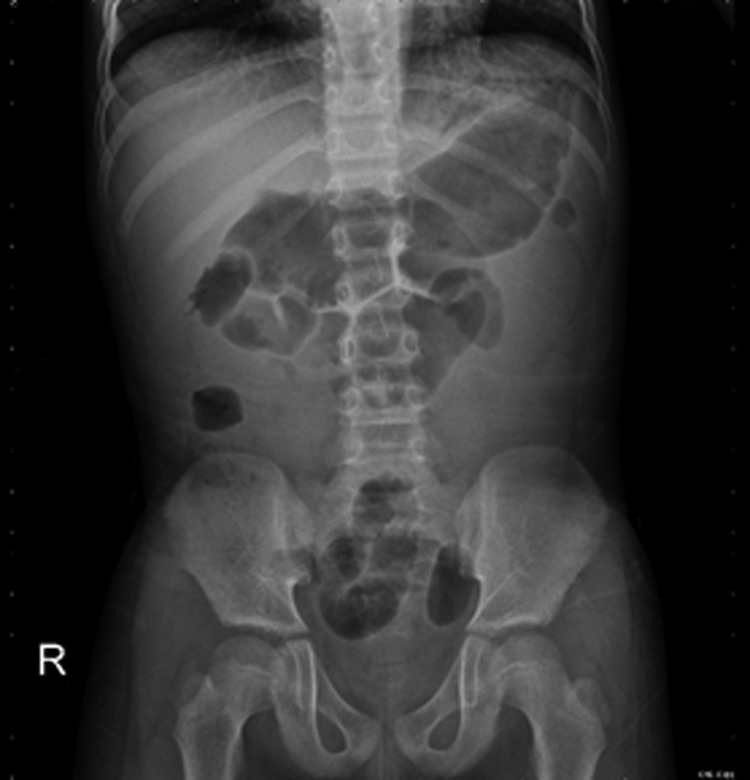
Kidney, ureter, and bladder studies revealed increased stomach and bowel gas patterns without obvious ureteral calculi.

**FIG. 4.**
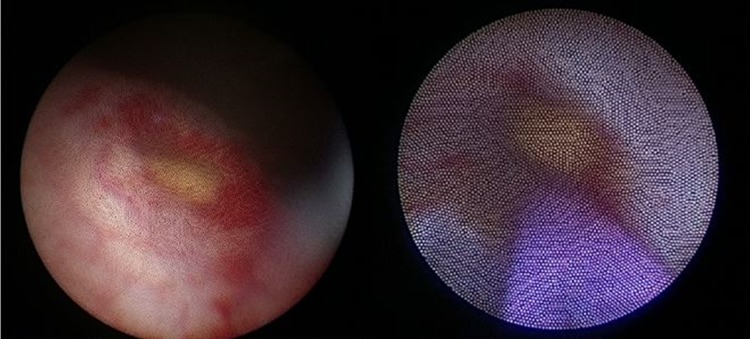
Right ureteral stone with inflammation at the orifice and left ureteral stone ∼2 cm proximal to the ureterovesical junction.

However, during follow-up at 10 days postoperatively, the patient complained of mild abdominal discomfort with poor appetite and reported vomiting twice. No fever was reported, and his wound was dry, clean, and healing well. Physical examination revealed knocking pain over the bilateral flank area. Follow-up with abdominal ultrasonography revealed bilateral hydronephrosis, but no intra-abdominal abscess (Fig. 2). Laboratory analysis revealed a white blood cell count of 13,900 μL (range: 3500–9100 μL; neutrophilia 82.5%) and creatinine levels of 12.85 mg/dL (range: 0.70–1.30 mg/dL). Oliguria was also noted. Kidney, ureter, and bladder radiograph (KUB) studies revealed increased stomach and bowel gas patterns, but no obvious ureteral stone formation (Fig. 3). Because bilateral ureteral obstruction with hydronephrosis was suspected, cystoscopy was conducted. Bilateral obstructing stones were noted at the right ureteral orifice and left ureter, ∼2 cm proximal to the ureterovesical junction, resulting to severe hydronephrosis (Fig. 4). We used flexible 4 mm ureteroscope and forcep for extracting the stones. No ureteral meatotomy or laser was used. Bilateral Double-J catheters (F 4.7 × 24 cm) were inserted. Postoperation, KUB follow-up revealed that the right Double-J catheter was appropriately placed; however, it was observed to be kinking at the ureter. Laboratory analyses conducted on postoperative day 2 revealed a white blood cell count of 12,300 μL (range: 3500–9100 μL; neutrophilia 84.9%) and creatinine levels of 0.76 mg/dL (range: 0.70–1.30 mg/dL). Kidney sonogram revealed mild left hydronephrosis. The bilateral Double-J catheter was removed 1 month after outpatient follow-up, and only a mild blood clot was noted at the right ureter. Furthermore, the kidney sonogram revealed no hydronephrosis.

## Discussion

This report presents an unusual case of bilateral hydronephrosis caused by ureteral calculi after LA in a child. Few studies have reported bilateral ureteral obstruction with anuria and acute renal insufficiency in children aged 6–15 years.^1–4^ Complications were generally noted between 3 and 16 days after appendectomy. Patients effectively recovered after ureteral stent insertion. These complications seem unrelated to appendix perforation. Hugen and colleagues reported five children with bilateral ureteral obstruction after appendectomy for a perforated appendix.^1^ van Linde and coworkers reported the case of a child with bilateral ureteral obstruction noted 4 days after appendectomy for perforated appendicitis.^2^ However, Timm and associates reported bilateral distal obstruction a few days after nonperforated appendectomy.^3^ Gupta and colleagues reported two pediatric patients with bilateral ureteral obstruction after open appendectomy: one with perforated appendix and the other without.^4^ The relationship between ureteral complications after appendectomy needs to be further investigated.

Several studies have reported postappendectomy right ureteral damage; however, postappendectomy ureteral obstruction is rare. We analyzed case reports published between 1995 and 2018 (Table 1), wherein 20 children (all males) experienced bilateral ureteral obstruction after appendectomy. Interestingly, all 20 had bilateral ureteral complications rather than only right ureteral complications. There are several hypotheses for this observation. Seeberg and coworkers^5^ reported that bilateral ureter obstruction may be caused by mechanical obstruction due to abscess. Hugen et al. assumed that localized peritoneal reaction caused by intraoperative bacterial contamination causes ureteral edema and obstruction predominantly in boys because their appendix is situated closer to the bladder, whereas internal genitalia separates the appendix and bladder in girls.^1^ Timm and colleagues supported the hypothesis that gangrenous or perforated appendicitis causes inflammatory changes to the posterior bladder wall, thus resulting in bilateral ureteral obstruction.^3^ We also support this hypothesis.

**Table 1.** Ureteral Obstruction after Appendectomy

*Ref.*	n	*Age*	*Gender*	*Location*	*Duration to obstruction (days)*
Grande et al.^6^	1	14	M	Bil.	9
Gupta et al.^4^	2	6, 11	M	Bil.	5, 3
Seeberg et al.^5^	1	11	M	Bil.	5
Nanni et al.^7^	1	6	M	Bil.	5
van Linde et al.^2^	1	6	M	Bil	4
Timm et al.^3^	1	11	M	Bil.	–
Green et al.^8^	8	6–15	M	Bil.	6–16
Hugen et al.^1^	5	9–15	M	Bil.	–

Bil., bilateral.

Only one study reported bilateral ureteral stone formation after appendectomy. In 2015, Grande et al.^6^ presented the case of a 14-year-old boy with necrotic appendicitis after LA, which was complicated with bilateral ureteral obstruction caused by millimetric stones on both distal ureters. Cystoscopy revealed inflammatory changes in the bladder base and a right-sided whitish plug protruding from the orifice. Multiple millimetric soft stones were removed using a 6F. semirigid ureteroscope with an endoscopic basket. Ureteral stricture was noted on the left distal ureter, ∼2 cm above the ureteral orifice. The urine sample showed no bacterial growth. Our patient's results were markedly similar to the results observed in this boy. Right-side ureter orifice millimetric stones were also noted, and ureter stents were placed after stone extraction. We also observed a left-side ureteral stricture with millimetric stones, ∼2 cm above the ureteral orifice. The pathophysiology of stone formation in these patients was unknown. Further data collection and analysis regarding this complication are warranted.

## Conclusion

In children, bilateral ureteral stone formation after LA is a rare complication. Surgeons must be aware of this complication when oliguria or hydronephrosis is observed. Early intervention is crucial to prevent irreversible renal damage. Extraction or using an endoscopic basket is both useful for stone removal. Bilateral ureteral stents placed for 2–4 weeks may be an appropriate treatment of choice.

## Abbreviations Used

CT: computed tomography
KUB: kidney, ureter, and bladder radiograph
LA: laparoscopic appendectomy

## References

[1] Hugen CA, Mulders PF, Monnens LA, de Vries JD Complete bilateral distal ureteral obstruction after appendicectomy. Lancet 1994;344:618–619 7980794

[2] van Linde ME, van Pinxteren-Nagler E, Klinkert P, de Jong TP, Schröder CH Acute renal insufficiency caused by bilateral ureteral obstruction after appendectomy in a 6-year old boy. Ned Tijdschr Geneeskd 2000;144:754–756 10812444

[3] Timm K, Illi OE, Leumann E, Stauffer UG Postrenal anuria after appendectomy in childhood. Eur J Pediatr Surg 1997;7:237–238 9297521 10.1055/s-2008-1071101

[4] Gupta V, Yadav SK, Al Said A Post appendectomy acalculus bilateral ureteric obstruction: A rare entity in children. Afr J Paediatr Surg 2013;10:377–378 24469492 10.4103/0189-6725.125453

[5] Seeberg LT, Edenberg J, Saetren H Bilateral ureteral obstruction after appendicectomy. Surgeon 2005;3:45–47 15789795 10.1016/s1479-666x(05)80012-5

[6] Grande M, Lisi G, Bianchi D, et al. Bilateral ureteral obstruction in children after appendectomy. Case Rep Surg 2015;2015:740795 26295001 10.1155/2015/740795 PMC4532893

[7] Nanni L, Vallasciani S, Valeri S, et al. Bilateral distal ureteral obstruction: unusual complication of appendicular abscess [in Spanish]. Cir Pediatr 2001;14:168–170 12601966

[8] Green JT, Pham HT, Hollowell CP, et al. Bilateral ureteral obstruction after asymptomatic appendicitis. J Urol 1997;157:2251 9146634

